# Cocaine Anæsthesia

**Published:** 1887-09

**Authors:** 


### Cocaine Anæsthesia.

Dr. Franz Fux, of Laibach, in an article on this subject, published in *Memorabilien*, May 10th, 1887, says:

Among the advantages of a local anæsthetic may be mentioned its entire freedom from danger to the life of the patient, although this danger has now happily been reduced to the minimum in etherization. This is especially true in regard to operations about the respiratory tract, as in tracheotomy. Again, a local anæsthetic can be administered without an assistant. This is of great importance to the country practitioner, who finds it often difficult or impossible to get the required number of skilled assistants to properly give the anæsthetic. So much has been written about the account of the introduction of cocaine that it is not necessary to review all the facts. Schroff, in 1862, first called attention to the astonishing effect of this drug. As soon as Prof. Wolfler made known the anæsthetic power of injections a great number of ways in which it was used successfully appeared in the medical journals.

The best way to apply cocaine for surgical purposes is as follows: Hydrochlorate of cocaine should be used, as cocaine itself is very insoluble, and the other preparations do not possess the same properties. Even if the drug be dissolved in twice distilled water the solution soon becomes turbid. This can be avoided by the addition of a little glycerine or of the bichloride of mercury. But the hydrochlorate is so soluble in water that no time is lost in preparing a fresh solution for each operation. There has been much discussion as to the strength of the solution to be used, and the number of points at which the solution should be introduced. Not less than three-quarters of a grain, nor over a grain and a half, should be used. If more than this is used cocaine intoxication may be produced. This can, however, be readily removed by the use of nitrite of amyl. From four to six injections may be made, rather than to use a weaker solution and make more injections. The injections should be made with a hypodermic syringe, and in a circle around the part to be operated upon, and they should go in underneath as far as possible. The syringe should be withdrawn gradually, and the last drop emptied as it nears the skin. It is well to wait from eight to ten minutes after the injection before beginning an operation. Anæsthesia produced in this way lasts from fifteen to twenty minutes, but this period may be prolonged by keeping the part moist with sponges soaked in a cocaine solution.

Cocaine suppositories are useful in spasm of the sphincter ani, and a five per cent. solution may be injected into the bladder before making an examination of the viscus, or in cases of painful visical catarrh.—*Med. and Surg. Reporter.*

